# Trends in the Design of Intensity-Based Optical Fiber Biosensors (2010–2020)

**DOI:** 10.3390/bios11060197

**Published:** 2021-06-15

**Authors:** Nerea De Acha, Abián B. Socorro-Leránoz, César Elosúa, Ignacio R. Matías

**Affiliations:** 1 Department of Electrical, Electronic and Communication Engineering, Public University of Navarra, E-31006 Pamplona, Spain; nerea.deacha@unavarra.es (N.D.A.); cesar.elosua@unavarra.es (C.E.); natxo@unavarra.es (I.R.M.); 2 Institute of Smart Cities, Public University of Navarra, E-31006 Pamplona, Spain; 3 Navarra Institute for Health Research (IdiSNa), Recinto de Complejo Hospitalario de Navarra, C/ Irunlarrea, 3, E-31008 Pamplona, Spain

**Keywords:** biosensor, optical fiber sensors, luminescence, fluorescence, absorption, localized surface plasmon resonance

## Abstract

There exists an increasing interest in monitoring low concentrations of biochemical species, as they allow the early-stage detection of illnesses or the monitoring of the environment quality. Thus, both companies and research groups are focused on the development of accurate, fast and highly sensitive biosensors. Optical fiber sensors have been widely employed for these purposes because they provide several advantages for their use in point-of-care and real-time applications. In particular, this review is focused on optical fiber biosensors based on luminescence and absorption. Apart from the key parameters that determine the performance of a sensor (limit of detection, sensibility, cross-sensibility, etc.), other features are analyzed, such as the optical fiber dimensions, the sensing set ups and the fiber functionalization. The aim of this review is to have a comprehensive insight of the different aspects that must be taken into account when working with this kind of sensors.

## 1. Introduction

In recent years, great efforts have been made to enhance our quality of life. Although most of them are devoted to the treatment or curing of severe diseases [1], their prevention and early detection are not only gaining interest among scientists [2] and companies [3], but also governments [4]. These goals involve, for instance, the real-time monitoring of water and air quality [5] or the rapid detection of biomarkers related to illnesses [6]. In order to meet these requirements, accurate, highly sensitive and fast biosensing devices have been developed using different technologies. Among them, one of the most studied during the last decades is that based on optical fiber. It offers several advantages such as biocompatibility [7], the possibility of in-situ monitoring [8] and the possibility of multiparameter sensing [9], among others. These features facilitate its utilization in point-of-care and/or real-time applications.

Optical fiber-based biosensors allow monitoring two main parameters: wavelength shifts and intensity variations [10,11]. Biosensing devices characterized by the first parameter were deeply analyzed in [12]. That contribution showed the three main ways to design wavelength-based optical fiber biosensors developed during last decade, as well as the benefits of working with wavelength-shift measurements. On this occasion, authors have considered it of great interest to bring together the latest advances in the field of intensity-based optical fiber biosensors within this contribution. In this manner, readers can have a broad view of the degree of development and possible applications of optical fibers as biosensing platforms.

Well-established existing technologies, such as ELISAs, fluorescence microscopy, DNA chips or flow cytometry, seem to solve the current needs in clinical analytics. Despite wavelength shifts, colorimetric techniques obtaining the results after a few minutes or strip-based detections where distinguishing the symptoms at first sight (normally by using colors) and then disposing of the active part are more easy-to-handle and more familiar to what is currently used in clinical analytics. In this sense, since intensity-based optical fiber techniques detect color changes and their use is really close to the day-by-day instrumentation in these areas, they constitute an interesting alternative to wavelength-based techniques that should not be underestimated at all. There are still some drawbacks to solve. For instance, the instabilities of the light source while providing the optical power can affect the assays, since they can mask an eventual enhancement of the obtained measurements. However, this is something that can be avoided by normalizing the measurements [13]. Other parameters that can also affect the measurements are temperature, relative humidity, light coupling from the sensor to the photoreceptor or the influence of ambient light.

The implementation of intensity-based optical fiber biosensors is quite simple. As it is well-known, every biosensor owns a substrate to guide the detection phenomenon, a bioreceptor to detect the target analyte uniquely and a biofunctionalization interface to attach the bioreceptor to the substrate. In the case of the technology presented in this contribution, the substrate is the fiber itself as a transporting waveguide. This simplifies the optical set-up a lot, since there is no need for dealing with extra material that involves, for instance, fusing fibers or depositing nanofilms onto the fibers to generate resonant phenomena. As long as the evanescent field of the optical fiber is able to reach the bioreceptor-analyte interaction, the main focus is to biofunctionalize the fiber properly and then detect the target analyte. Moreover, the employed instrumentation is simple and cost-effective. Since the measurements are taken at a specific wavelength, just a simple light source (i.e., an LED) and a photodetector centered at that wavelength are needed, no matter if the light is going to be measured using either transmission or reflection set-ups.

In view of the previous considerations, this bibliographic review will focus on those optical fiber biosensors that measure intensity variations. As it will be demonstrated, the utilization of these techniques for the detection of biomolecules is widespread [14,15], taking advantage of the improvements that optical fiber technology has experienced in the last decade. To this purpose, a first section describing the main operation principles used to detect phenomena based on intensity variations (luminescence and absorption, essentially) will be addressed. Then, the optical instrumentation and configurations that are commonly employed will be revised, going through their most relevant applications. Next, the sensors performance will be analyzed in detail in terms of their limits of detection (LOD), working ranges, sensitivities, cross-sensitivities and regeneration. This information will allow the reader to have a complete overview of the most important features of luminescence and absorption-based optical fiber biosensors, something that will be summarized in the conclusions section.

## 2. Intensity-Based Optical Detection Phenomena

**Figure 1:**
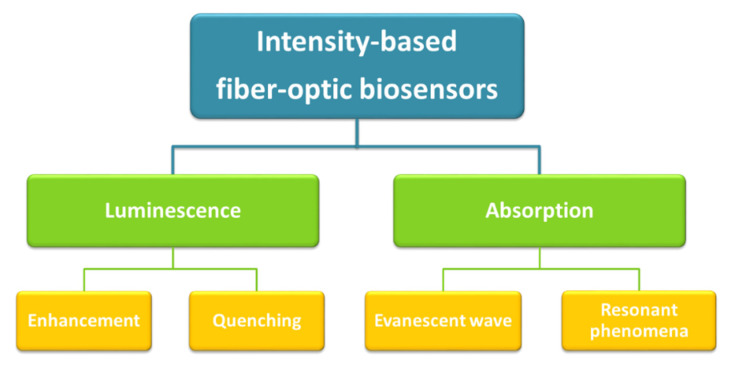
Schematic of the intensity-based optical fiber biosensors analyzed in this review.

Biosensors are devices that combine specific bioreceptors (e.g., antibodies, enzymes, or DNA strands) with a transducer (in the case of this review, the optical fiber) so that the interaction between the target analyte and the bioreceptor induces a change in the measured magnitude [16]. In the case of intensity-based optical fiber sensors, that magnitude is the intensity of the light transmitted through the fiber, which can be measure using two different detection methods, mainly luminescence and absorption, which are shown in Figure 1. The first one comprises those sensors based on luminescence, either when the light collected increases or decreases at a certain wavelength as a function of the analyte concentration. The second phenomenon is absorption. This will include those sensors that take advantage of the absorbing properties of the thin films deposited onto the fiber to detect what happens at a certain wavelength, or spectral range. Among the several advantages of employing thin films, the possibility of tailoring their features (selectivity, sensitivity, permeability) in the nanoscale [17], their easy fabrication and characterization [18] or the possibility of functionalizing them [19] are the most remarkable ones. They will be discussed in the corresponding section.

A common characteristic to every biosensor based on these techniques is the fact of working with multimode fibers. The first reason to justify this is that intensity-based sensors usually need a higher light intensity than phase modulated sensors [20]. In this sense, multimode fibers allow transporting high intensities along their thicker core. Moreover, it is possible to increase the light coupling by manufacturing different geometries on the optical fiber. The most representative ones are described throughout this section.

The second reason is the optical working range, since most of the reported biosensors work on the UV-VIS range. Apart from that, the use of molecular labels in the case of luminescence-based biosensors, which emit in the visible range, needs a low-loss waveguide to where to couple and transport the luminescent emission produced.

### 2.1. Luminescence

**Figure 2:**
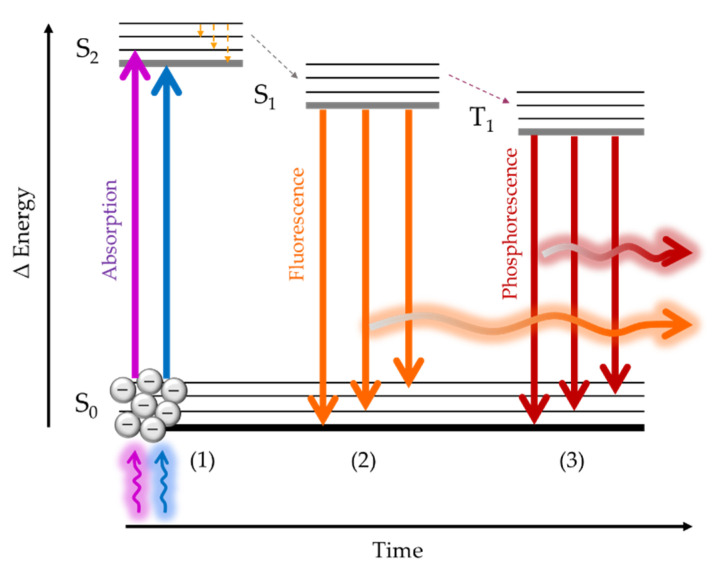
Schematic of a Jablonski’s diagram showing the two kinds of luminescence.

Luminescence is a well-established detection mechanism in different biomedical research areas. In fact, it is one of the most important sensing procedures in clinical and biological applications. It can be found when performing PCRs [21], ELISAs [22], fluorescence microscopy [23] or gene expression in DNA chips [24], among others [25]. Its working principle is based on the Jablonski’s diagram schematic shown in Figure 2. There are certain substances, called luminophores, that absorb the energy provided by photons with wavelengths located at the blue, violet and UV part of the electromagnetic spectrum. This energy is absorbed by the existing electrons in the different orbitals, which are promoted to higher energy orbitals in a process called “excitation” (1). Then, since the electrons tend to return to their lowest energy state, they will progressively go back to their original orbitals (2), thus emitting part of the energy they have absorbed in the form of lower energy photons (3). This means at wavelengths located within the green to red and even NIR spectrum [26].

These transitions from the excited states to the ground states are called radiative transitions. However, there also exist certain electron transitions where light emission does not take place. They are the non-radiative transitions (dashed arrows in Figure 2), which occur through several mechanisms, such as vibrational relaxation, intersystem crossing or internal conversion [27]. Both radiative and non-radiative transitions influence the quantum efficiency, this means the ratio between the absorbed photons and those emitted as luminescence. This is another important parameter that determines the behavior of a fluorophore and, therefore, its choice when performing luminescence-based measurements.

For radiative transitions, the duration of the emission time is known as lifetime, and it allows distinguishing between fluorescence (lifetimes from 10^−10^ to 10^−5^ s) and phosphorescence (lifetimes from 10^−4^ to 10^4^ s). The wavelength range between the emission and the absorption peaks is known as Stokes shift. The larger it is, the simpler is the experimental set-up required, as there is no need for utilizing optical filters. However, when working with continuous light sources, if both peaks are close, optical filters are necessary in order to isolate the excitation from the emission light at the photoreceptor.

Furthermore, the luminescent intensities and lifetimes of some luminophores depend on the presence of certain analytes or on the conditions of the surrounding media [28], so they are of great interest for the development of optical biosensors. This can be done by attaching the luminophores to the bioreceptor [29,30] or to the target analyte [31]. In some cases, it is the binding between the bioreceptor and the analyte that induces changes in the surrounding medium of the luminophore [28]. This normally leads to quenching or enhancement processes (i.e., shorter or longer lifetimes). Another possibility is to label the analyte with a luminophore, so when it joins the bioreceptor, the luminescent emission can be captured. Therefore, luminescence quenching as well as luminescence enhancement biosensors can be developed. To the first group belong all the sensors whose intensity decreases in the presence of the target analyte. In the second case, the opposite phenomenon takes place: the luminescent emission increases due to the presence of the target analyte.

**Figure 3:**
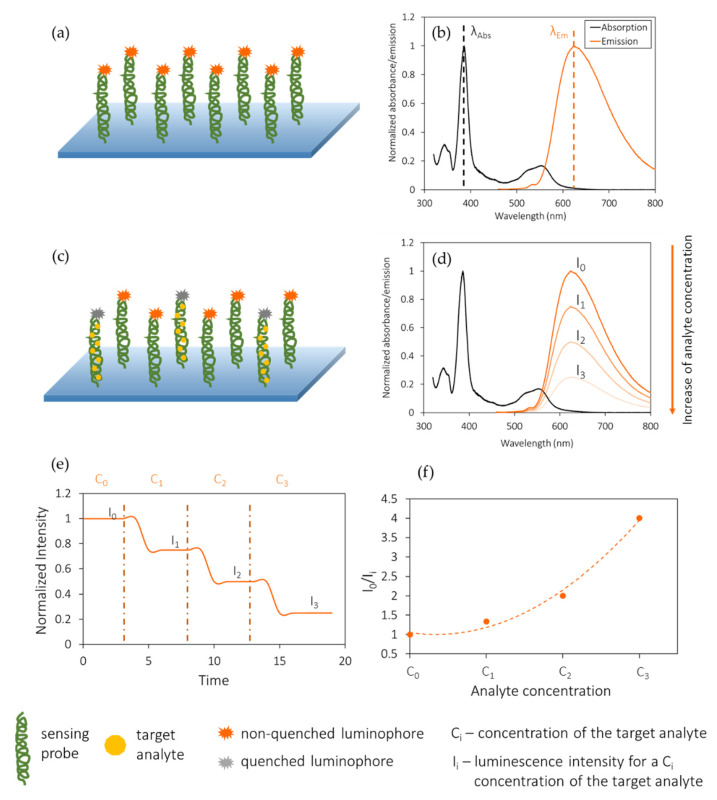
Working principle of a luminescence quenching-based optical fiber biosensor. (**a**) Fiber functionalized with a luminophore-labelled bioreceptor. (**b**) When illuminated at the absorption wavelength of the luminophore, luminescent emission takes place. (**c**) Quenching of the luminophore in the presence of target analyte. (**d**) Decrease in luminescent emission as the target analyte concentration increases. Dynamic response (**e**) and calibration curve (**f**) of the biosensor. The purpose of this schematic is to show the generic behavior of this kind of sensors, so the graphs do not correspond to experimental or simulated data, nor to any existing material.

Figure 3 shows the working principle of a luminescence quenching-based optical fiber biosensor. The fiber is first functionalized with a luminophore-labelled bioreceptor (a) that is illuminated at the absorption wavelength of the luminophore (b). As the target analyte concentration (C_i_) increases, the luminophores are quenched (c), so the emitted intensity (I_i_) decreases (d). The dynamic response of the sensor is depicted in Figure 3e, whereas the obtained calibration curve is shown in Figure 3f. In the case of luminescence enhancement sensors, the opposite process occurs: as the analyte concentration increases, so does the luminescent emission. Only the first process is shown for simplicity.

As it has been explained, the luminescence emission by the luminophores occurs due the absorption of energy in the form of light, which requires their exposure to a light source. If this exposure is long-term, it can lead to a photochemical modification of the luminophore, inhibiting the transition of the electrons from the ground state to the excited ones, and, in consequence, decreasing the emitted number of photons. This phenomenon is known as photobleaching and, although it can be mathematically modeled and compensated [13], its effect must be reduced as much as possible because it damages the structure of the luminophore [32,33].

Among the different elements that can be used as bioreceptors, aptamers (chemically synthesized DNA or RNA strands or oligonucleotides) [34], DNA strands, proteins and antibodies are the most common. Regarding the applications of these sensors, DNA [35], glucose [36] and metal ions detection [37] are the most widespread. Despite them, other bioanalytes such as proteins [38] or bacteria [30] can also be detected.

The performance of luminescence quenching-based sensors are usually modeled by the Stern-Volmer equations, as they allow the analysis of the distribution of the luminophore inside the sensing film. When it is homogeneously distributed, the quencher affects the whole luminophore population equally, so the calibration curve follows a linear tendency, given by the Stern-Volmer Equation (1) [26]:

(1) $$\frac{I_{0}}{I}=1+K_{SV}\cdot \left[Q\right]$$

where [*Q*] represents the concentration of the target analyte (quencher), *K_SV_* is the quenching constant, *I*_0_ the luminescent intensity for 0% of quencher concentration and *I* the luminescent intensity for a given analyte concentration.

In the cases in which the luminophore population is heterogeneously distributed, the calibration curve *I*_0_/*I* follows the Demas model [39] according to Equation (2):

(2) $$\frac{I_{0}}{I}={\left(\frac{f_{1}}{1+K_{SV,1}\cdot \left[Q\right]}+\frac{f_{2}}{1+K_{SV,2}\cdot \left[Q\right]}\right)}^{-1}$$

where $f_{1}$ and $f_{2}$ ($f_{2}=1-f_{1}$) correspond to the populations of the fluorophore, and $K_{SV,1}$ and $K_{SV,2}$ to their quenching constants, respectively.

These equations are of great utility when analyzing luminescence-based systems, as they allow the investigation of bioreceptor and quencher distribution, association, diffusion and reaction at the molecular level [40].

### 2.2. Absorption

**Figure 4:**
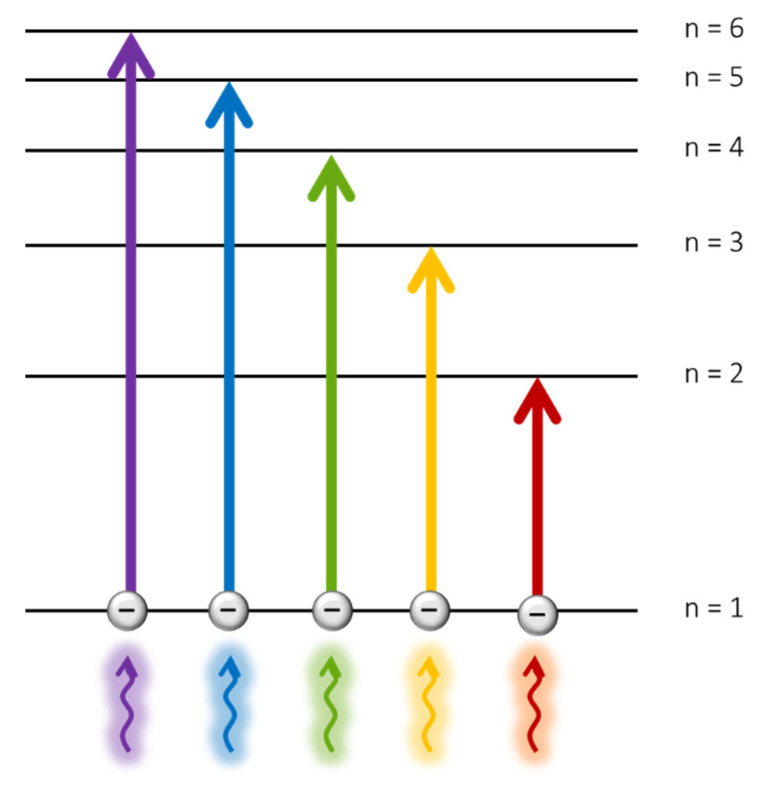
Schematic of an absorption-based technique. Incident photons promote electrons to higher level orbitals, leading to absorption/transmission phenomena as a function of the analyte increasing concentration.

Absorption-based sensing has been commonly employed in a wide variety of applications, ranging from gas detection [41] to water-quality monitoring [42], but also biodetection [43]. The transduction principle is determined by the energy levels of the different materials: as electrons can only exist in discrete energy levels, to be moved from the ground state to higher energy levels, they must absorb enough energy, which is acquired from the absorbed light, as it is depicted in Figure 4. Thus, the absorption spectrum of a certain material represents the wavelengths at which light provides to its electrons enough energy to reach higher energy levels.

There are several ways of detecting the presence of analytes using this technique. However, it is important to classify them according to how the absorption takes place. A first group of absorption-based sensors is that in including a selective group of substances, which due to their internal structure, conformation, polarization or even their own chemistry or physics, they can absorb light at a specific wavelength. This is the case of substances such as acetone, ozone, sulfur dioxide or transition metals, which present an absorption band within the UV range [44,45]. Their detection can be carried out with no sensitive material coatings onto the substrate (i.e., the optical fiber) [46].

A second group is comprised of those sensors that need the functionalization of a substrate with a bioreceptor or a sensing film onto it [47]. They are often based on evanescent wave absorption (EWA), which consists of the interaction of part of the light transmitted through the substrate with the surrounding medium [48]. Specifically, the evanescent wave is modified by changes on the refractive index of the surrounding medium, which increases or decreases the light coupled to the cladding modes of the fiber substrate. A critical factor here is the penetration depth of the evanescent wave (d_p_). If d_p_ reaches the bioreceptor-analyte area, it will be possible to detect the magnitude of the biological interaction. In this case, the presence of substances that absorb light in the range of the working wavelengths will cause a reduction in the transmitted intensity.

The last option is also based on EWA, but it has been considered apart due to the recent developments and high number of contributions recently published. In some specific cases, optical fiber structures designed to detect wavelength shifts are used as absorption-based biosensors. That is the case of Localized Plasmon Surface Resonances (LSPR) using metallic nanoparticles (NPs), usually made of gold (Au) or silver (Ag) [49]. These NPs are of great interest as they can be biofunctionalized with a specific bioreceptor to detect the target analyte [50]. In these cases, once the LSPR is located at the desired wavelength, the univocal bioreceptor—analyte interaction induces more or less absorption [12] as a function of the increasing analyte concentration, allowing the characterization of this kind of biosensors.

**Figure 5:**
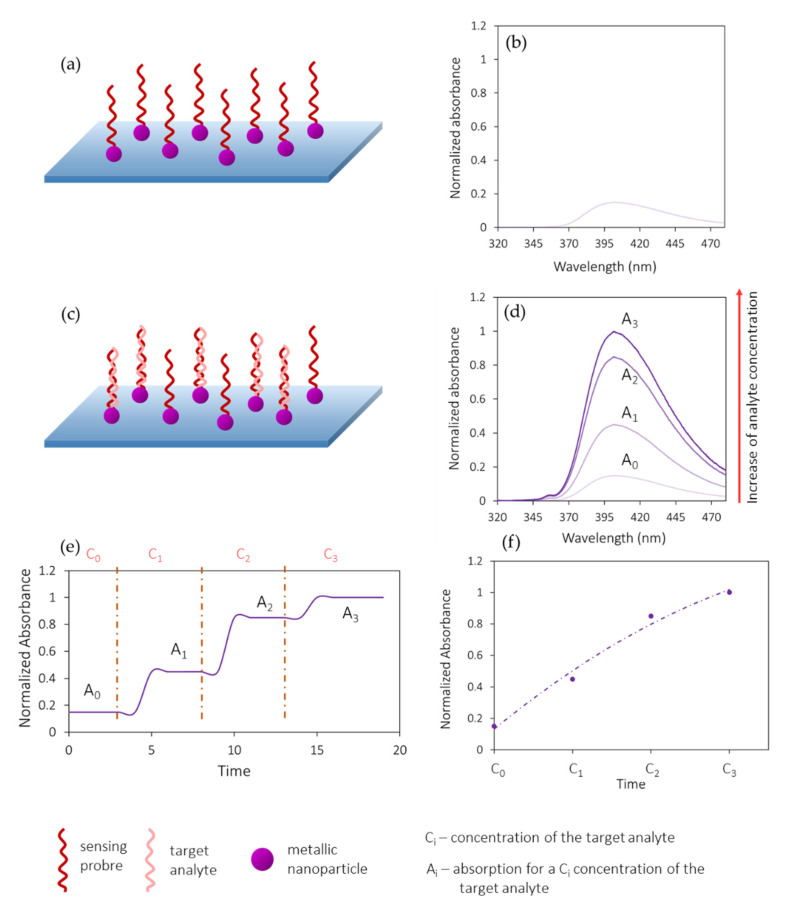
Working principle of an absorption-based optical fiber biosensor. (**a**) Fiber coated with a metallic nanoparticle-labelled bioreceptor. (**b**) Original absorption spectrum centered at a determined wavelength. (**c**) Increasing bioreceptor—analyte binding, which increases the absorption at that wavelength (**d**). (**e**) Dynamic response and (**f**) calibration curve of the optical fiber biosensor. The purpose of this schematic is to show the generic behavior of this kind of sensors, so the graphs do not correspond either to experimental or simulated data, or to any existing material.

Figure 5 shows the working principle of an absorption-based biosensor developed using a metallic nanoparticle functionalized with a bioreceptor (a). This sensing probe presents an original absorption band centered at a determined wavelength (b). As the concentration of the target analyte increases (c), it binds the bioreceptor and this makes the absorbance increase (d), which can be taken into account to monitor the dynamic response of the biosensor as well as to obtain its calibration curve (e and f). This working principle can be applied to other absorption-based sensors that do not require a previous functionalization of the optical fiber. In those cases, the initial absorbance will increase as a function of the analyte concentration.

## 3. Optical Parameters Analyzed

The response of an optical fiber biosensor is influenced by the optical instrumentation and also by the number of correctly attached bioreceptors to the optical substrate and their performance. Regarding the optical instrumentation, the combination of the light source, the photodetector and the optical fiber geometries should discriminate minimum intensity variations, which increases the system resolution. In relation to the bioreceptors, they play a key role as they are responsible for the detection of the target analytes. An optimized combination of both of bioreceptors and instrumentation will determine the response of the optical biosensors, as it defines their parameters, i.e., the sensitivity, LOD, dynamic range, regeneration and cross-sensitivity. All of them are going to be analyzed in this section.

**Figure 6:**
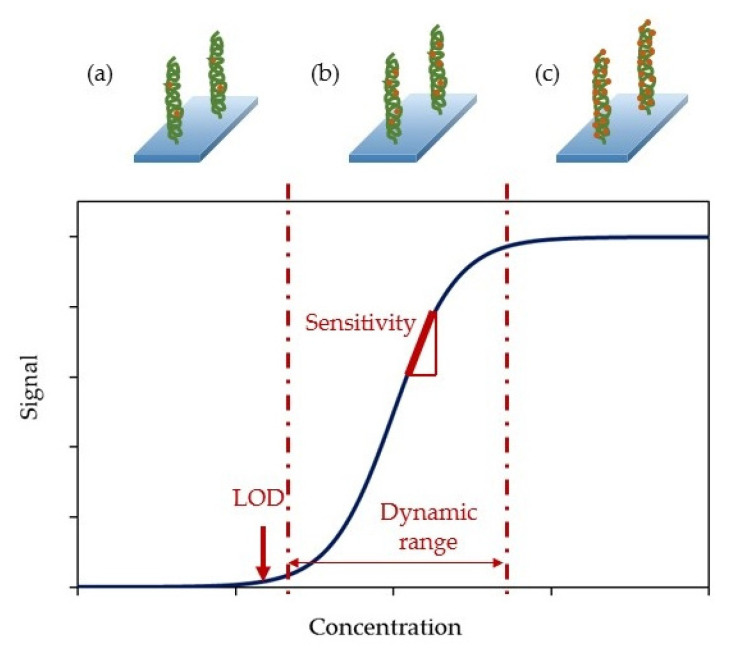
Sensitivity, limit of detection and dynamic range of a calibration curve. (**a**) The LOD is the minimum detectable concentration of the analyte, (**b**) the dynamic range is limited by the LOQ and the upper limit and (**c**) over the upper limit, the bioreceptor is saturated and it is not capable of detecting higher concentrations of the analyte.

The calibration curve of an optical intensity-based biosensor is a function that presents the evolution of the emitted or absorbed light by this biosensor as the analyte is progressively detected. As shown in Figure 6, after obtaining this graph it is possible to deduct three main magnitudes that can describe the performance of the biosensor: the sensitivity, the LOD and the dynamic range. These three together with other interesting parameters will be analyzed below, in order to establish the different classifications that will be made in the next section.

- Sensitivity: it is the slope of the calibration curve. That is, the ratio between the intensity (or absorbance) variation and the analyte concentration variation, typically measured at every point of the calibration curve. In the case of luminescence-based biosensors, the corresponding expression is given by (3), while for absorption-based biosensors, the intensity increment should be substituted by an absorption increment.
  (3) $$S=\frac{\Delta I}{\Delta \left[analyte\right]}$$
  while intensity and absorbance are always expressed in arbitrary units (AU), the analyte concentrations can be expressed in different units (mol/L, colony forming units (CFU), etc.) depending on the way the analyte concentration is measured. Moreover, the responses of the sensors tend not to be linear but sigmoidal. Normally, after taking the data it is typical to perform a data conditioning to logarithmic scales before calculating their sensitivities [51].
- Limit of detection (LOD): it is the lowest amount of analyte that can be detected but not necessarily quantitated by the sensor [52]. It is typically calculated as the mean of the values of the reference base line plus three times their standard deviation (4):
  (4) $$y_{LOD}={\bar{y}}_{blank}+3\sigma_{blank}$$
  where $y_{LOD}$ is the LOD of the sensor, ${\bar{y}}_{blank}$ corresponds to the average of the reference samples and $\sigma_{blank}$ is the standard deviation.
- Dynamic range: is the range of analyte concentrations between the limit of quantification (LOQ) and the upper limit. The first one is the minimum analyte concentration that can be detected and measured, while the second corresponds to the maximum concentration that the sensor can detect without being saturated.
- Cross-sensitivity: it is defined as its sensitivity towards other substances different from the target analyte [53]. As it is an indicator of the selectivity of a sensor, it is a key parameter when characterizing it.
- Resolution: it is the minimum change of the measured magnitude that can be detected [54].
- Detection media: apart from detecting biomolecules in standard conditions (i.e., ultrapure water or buffered solutions), biosensors should be capable of working in serum samples, real samples or, at least, solutions that mimic them.
- Regeneration: an effective manner of reducing the cost per test is the possibility of reusing the biosensors. Thus, regenerating the sensors surface has become of great interest and several mechanisms have been developed to reach this goal.

## 4. Classification and Discussion on Intensity-Based Optical Fiber Biosensors

### 4.1. Optical Fiber Biosensors Based on Luminescence

**Figure 7:**
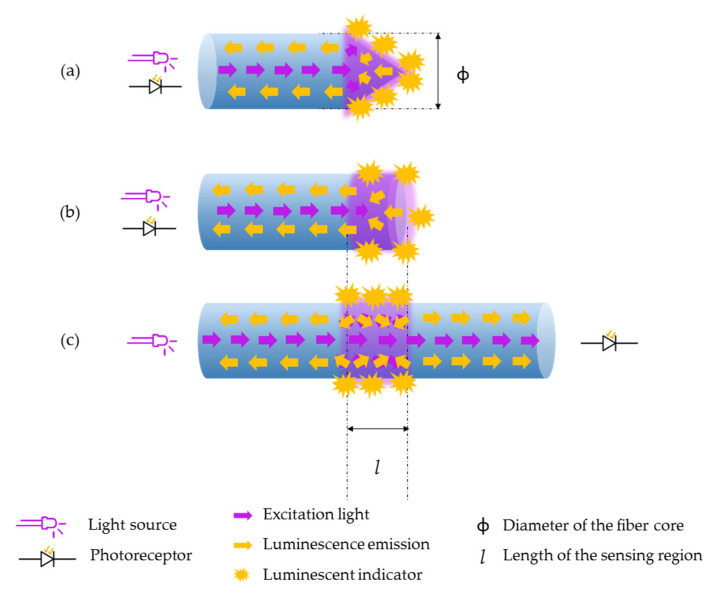
Different sensing schemes to fabricate luminescence-based optical fiber biosensors. (**a**) An optical fiber with a tapered tip increases its sensing surface gently (reflection architecture). (**b**) A perpendicular cut on the tip of an optical fiber is also used for biosensing using a reflection architecture. (**c**) In the transmission architecture, the sensing region is located along the fiber core.

Luminescent sensors monitor the concentration of the target analyte by measuring the intensity variations of the employed luminophore. Due to the reduced dimensions of the optical fiber, light coupling from the luminophore to the photoreceptor is critical [55]. Thus, several optical schemes are employed, although the reflection architecture is the most widespread [13]. In particular, tapering the tip of the optical fiber increases the area of the sensing surface, allowing to maximize the luminescence coupling and to minimize the reflection of the excitation light [56]. On the contrary, using a transmission set-up hinders the light coupling from the fiber to the sensing film as well as from the sensing film to the photodetector. In spite of that, some authors still use that architecture [57]. The previous three sensing schemes are depicted in Figure 7.

With the same goal of increasing the interaction area between the probe and the analyte, multimode fibers (made of silica or a polymeric material) are used as substrates when fabricating these sensors. The typical fiber diameters employed range from 400 μm to 1.96 mm [35,58]. Lower fiber core diameters are less common, but are still used [59].

**Figure 8:**
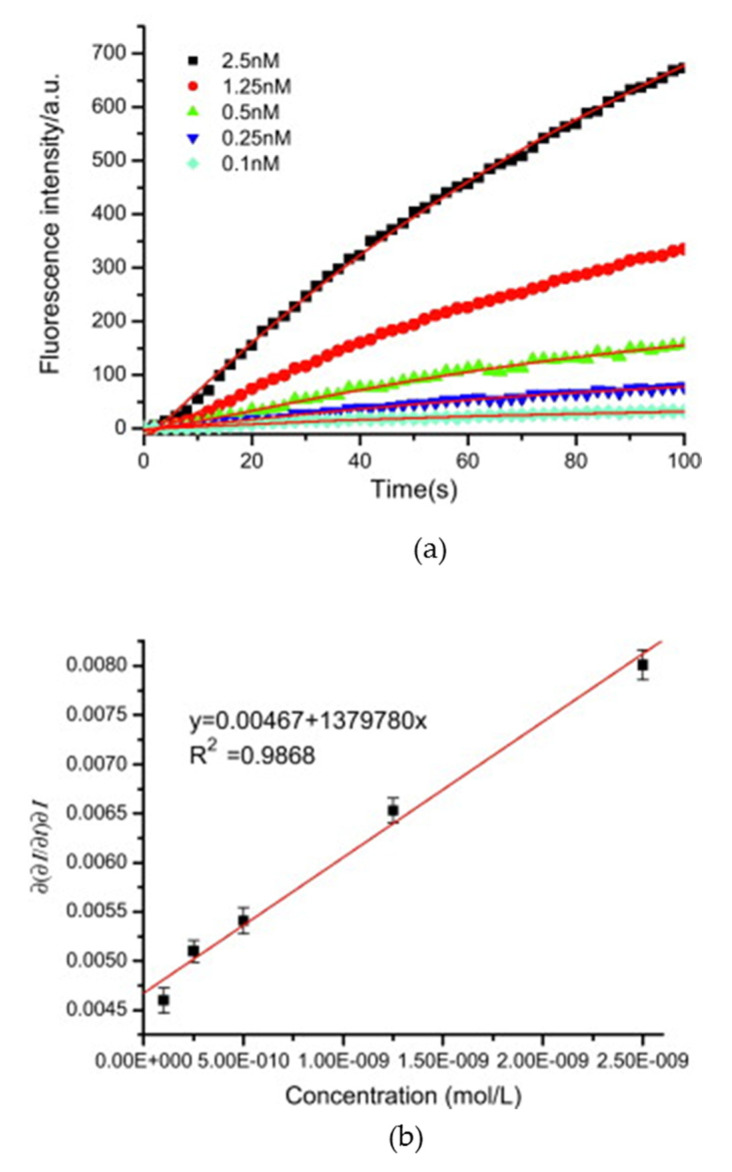
(**a**) Kinetic curves representing the molecular interactions on the sensor surface. (**b**) Determination of binding kinetics for DNA hybridization. Reprinted with permission from [31].

DNA detection is based on the high affinity shown by complementary DNA strands [60]. These sensors consist of the adsorption of the bioreceptor (i.e., a single-stranded DNA) which is complementary to the target DNA strand, onto the surface of the optical fiber [29,35]. For instance, Long et al. developed a biosensing platform capable of detecting 3.2 aM of the target DNA [31]: the single-stranded DNA that served as bioreceptor was immobilized onto a silanized 600 µm-core tapered optical fiber using streptavidin and the heterobifunctional cross-linker *N*-(4-maleimidobutyryloxy) succinimide (GMBS). The sensor was exposed to different quantum dot (QD)-labeled DNA strands: complementary DNA, one-base mismatched complementary DNA, and non-complementary DNA. Due to the hybridization between the DNA strand and the QD-labeled complementary DNA, the fluorescence intensity increased as the concentration of the complementary DNA did (Figure 8), while no fluorescent emission was detected in the presence of non-complementary DNA. Furthermore, the surface of the sensor was regenerated using sodium dodecyl sulfate (SDS) at pH 1.9, which allowed its reuse at least 30 consecutive times, with a decrease in the maximum fluorescence intensity lower than 8%.

Luminescent indicators can be attached to the bioreceptor instead of to the target DNA strand. It is so in the case of Giannetti et al. [29], where the molecular beacon 5′-(ATTO647N)GAGAAAGGGCTGCCA(Thiol)-3′ was immobilized onto a 600 µm-core tapered optical fiber. The sensor was exposed to a DNA sequence complementary to that acting as bioreceptor as well as to random DNA strands: the luminescent emission increased as the presence of the target DNA did. However, no variation of the luminescent intensity was observed in the presence of random DNA sequences.

**Figure 9:**
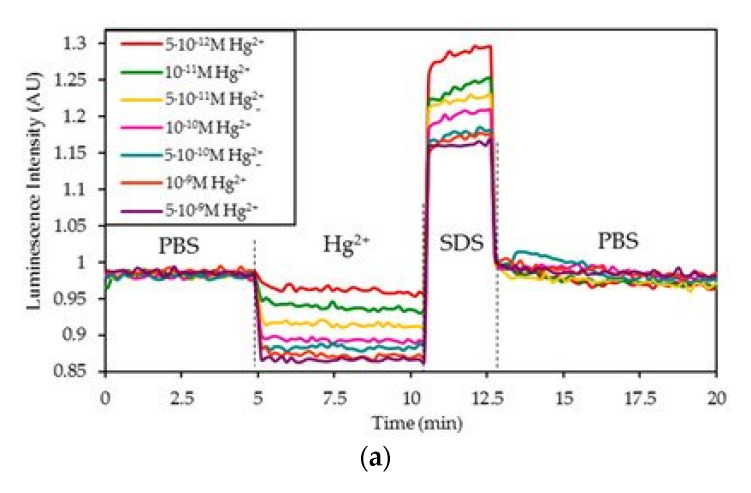

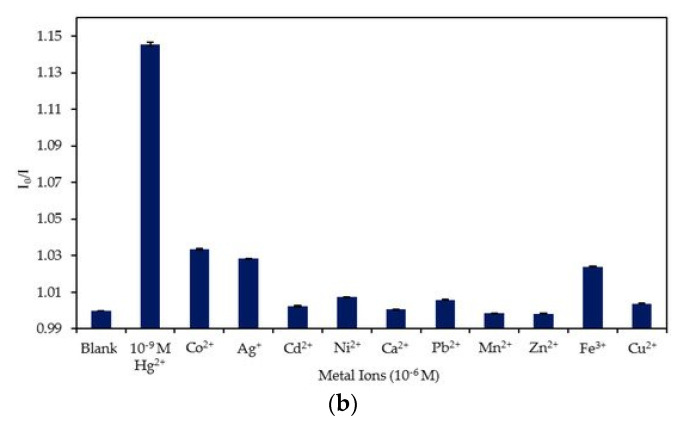
(**a**) Normalized luminescence variation for 5 × 10^−12^ M, 10^−11^ M, 5 × 10^−11^ M, 10^−10^ M, 5 × 10^−10^ M, 10^−9^ M and 5 × 10^−9^ M concentrations in 10 mM PBS (pH 7.4), and regeneration with 0.5% *w*/*w* SDS. (**b**) *I_0_/I* ratio of the sensor in the absence of metal ions (blank), in the presence of 10^−9^ M Hg^2+^ and in the presence of 10^−6^ M of Co^2+^, Ag^+^, Cd^2+^, Ni^2+^, Ca^2+^, Pb^2+^, Mn^2+^, Zn^2+^, Fe^3+^, and Cu^2+^ ions. Reprinted with permission from [51].

Apart from the detection of complementary DNA strands [61], the monitoring of other analytes, for instance, mercury (Hg^2+^) or lead (Pb^2+^) ions is also a widespread application of fluorophore-labeled DNA sequences [62,63]. In the first case, the detection of Hg^2+^ ions is due to the conformational change induced by these ions into thymine(T)-rich oligonucleotide (ON) sequences: in the presence of Hg^2+^ ions, T-Hg^2+^-T mismatches are formed [64], so the T-rich sequences acquire a hairpin structure [65]. Furthermore, those T-Hg^2+^-T base pairs quench the luminescent emission of the indicator labeled to the ON sequences [66]. For instance, the sensor described in [51] exhibited an LOD of 4.73 × 10^−13^ M Hg^2+^ ions in a phosphate buffered solution (pH 7.4). Due to the high affinity of Hg^2+^ ions to T bases, the sensor responded in less than 25 s for the highest Hg^2+^ concentrations in a reversible manner (Figure 9a), and it showed a low cross-correlation to other metallic ions (Figure 9b). The sensor also allowed the measurement of 5 × 10^−12^ M Hg^2+^ ions in ultrapure and tap water.

Aptamers present the advantage of being highly specific bioreceptors [67], so they are of great interest for the development of biosensors. Among many other applications, they can be employed for the detection of endocrine disrupting compounds, such as 17β-estradiol [68]. Taking advantage of this, N. Yildirim and co-workers [69] reported an optical fiber biosensor capable of detecting 17β-estradiol concentrations from 5 × 10^−9^ to 75 × 10^−9^ M, with a LOD of 2.1 × 10^−9^ M. Its regeneration was carried out with a 0.5% SDS solution (pH 1.9) for 90 s. Furthermore, the biosensor was not only tested in Tris-HCl buffer, but also in wastewater treatment effluent samples.

**Figure 10:**
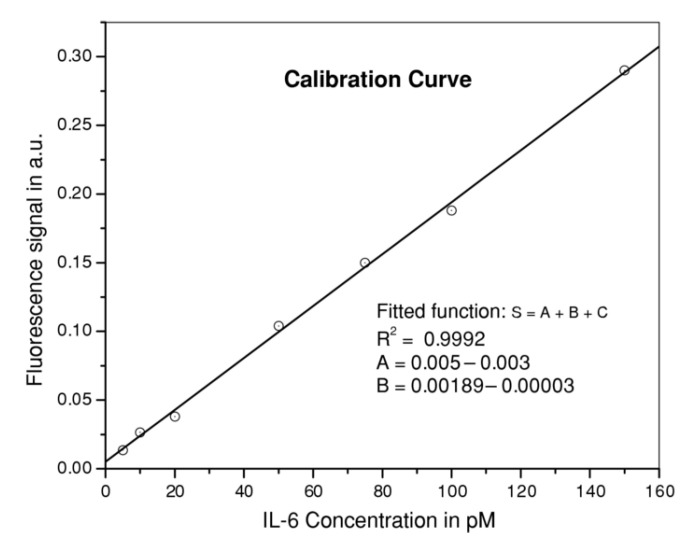
Calibration curve of the IL-6 sensor in the pM range. Reprinted with permission from [70].

Antibodies can be also labeled with fluorophores. Wang et al. detected up to 5.9 ± 0.6 pM concentrations of interleukin-6 (IL-6) in serum samples from lupus patients [70]. The sensor was developed by immobilizing Alexa Fluor 488–labeled anti-IL-6 antibodies onto the silanized surface of a 600 µm-core tapered optical fiber, using a reflection architecture. It showed a linear behavior in the pM range, as it can be observed in Figure 10, and a specificity of 100% in serum samples.

Highly sensitive and specific biosensors based on fluorophore-labeled antibodies can be achieved taking advantage of the combination of different optical techniques. It is the case of localized surface plasmon coupled fluorescence (LSPCF)-based sensors, which consist of the generation of an SPR with AuNPs, whose electromagnetic field excites the emission of the indicator labelled to the antibodies. An LSPCF optical fiber biosensor was developed by Chang et al. [71] utilizing protein A-adsorbed AuNPs and Atto633-labeled a-H1 antibodies, which were mixed and deposited along the core of a 1 mm-diameter plastic optical fiber. The sensor was based on the detection of hemagglutinin (HA) proteins, which contain the antigenic regions of the Swine-origin influenza A (H1N1) virus (S-OIV). The reported LOD was 13.9 pg/mL of HA, which is 103-fold lower than that obtained using the conventional capture ELISA, and its response was linear from 5 to 50 ng/mL. The sensor also improved the detection sensitivity of S-OIV up to 50-fold in PBS and 25-fold in mimic solution.

Proteins are also bioreceptors that can be used for the detection of other biomolecules [72]. It is the case for glucose binding protein (GBP), which has been widely employed for the detection of that analyte [58]. The binding of glucose to GBP induces conformational changes in that protein, which can be monitored with polarity-sensitive fluorescent probes. The one chosen by C. Tiangco et al. for the development of a glucose biosensor was 6-bromoacetyl-2-dimethylaminonaphthalene (BADAN): the authors immobilized BADA-labelled GBP onto Ni-NTA agarose beads for their deposition onto the tip of an optical fiber [36], fabricating a biosensor that was tested in vitro in PBS and in Yucatan minipig skin, which was used as surrogate for human skin. The sensor detected 2 × 10^−6^ M glucose in Yucatan minipig skin. Apart from that, it presented a reversible and repetitive (relative standard deviation of 4.65% for 6 μM and 7.04% for 10 μM glucose) behavior in PBS and a response time of 15 s.

The potential of luminescence-based optical fiber biosensors for real applications is shown in [73]. Here, a portable microarray biosensing platform based on four optical fiber sensors working in parallel was developed. Using optical switches, it was possible to illuminate all the fibers and to collect their fluorescent emission using just one photodiode and a single photodetector. Each of the fibers were functionalized using Cy5.5-labelled Microcystin-LR (MC-LR), 2,4-Dichlorophenoxyacetic acid (2,4-D), Atrazine (ATZ) and Bisphenol A (BPA) antibodies, respectively, in order to detect the four pollutants. In buffered solutions the sensors presented LODs of 0.04 μg/L, 0.09 μg/L, 0.02 μg/L, and 0.03 μg/L, respectively. Furthermore, the platform was also tested in real samples, where the presence of other molecules produced a negligible influence on the performance of the sensors. Apart from that, it was possible to regenerate their surfaces using 0.5% (*w*/*w*) SDS solutions.

The devices reported in this section, as well as many other applications developed during the last years in the field of luminescence-based optical fiber biosensors are summarized in Table 1, Table 2, Table 3 and Table 4 according to the type of bioreceptors and analytes: aptamers or DNA strands for the detection of other aptamers or DNA strands (Table 1), aptamers or DNA strands for the detection of other analytes (Table 2), antibodies as bioreceptors (Table 3) and other bioreceptors (Table 4).

### 4.2. Absorption-Based Optical Fiber Sensors

**Figure 11:**
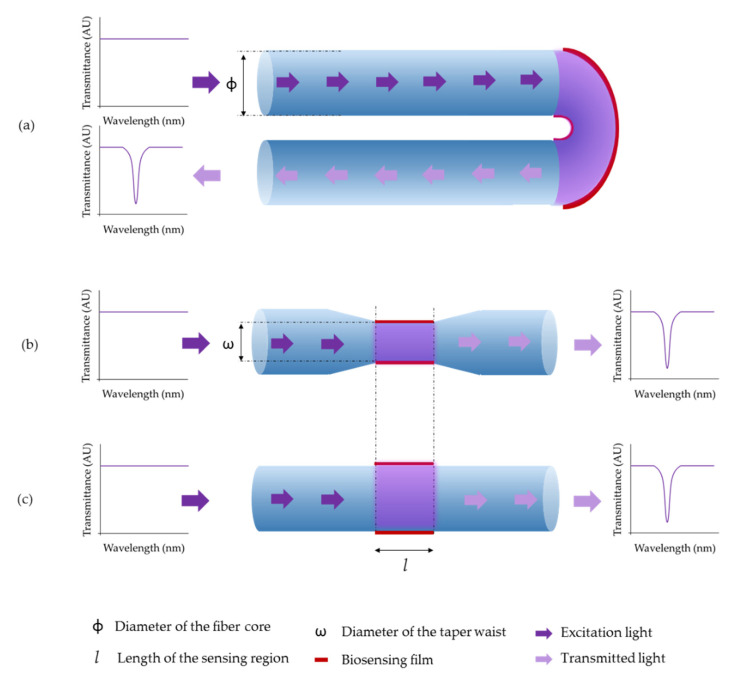
Different sensing schemes developed for absorption-based optical fiber biosensing: (**a**) the U-bent region of the optical fiber is coated with the sensing film, (**b**) the sensing film is deposited onto a tapered region of the fiber and (**c**) the sensing region is located along the fiber core.

As explained in Section 2.2, absorption-based optical fiber sensors use the interaction between the light transmitted through the optical fiber with the surrounding media to monitor the presence of the target analyte. A crucial parameter of these sensors is the penetration depth [91]. In this sense, in order to improve their sensitivity, several parameters of the optical fiber are typically tailored, for instance, the fiber core diameter, the fiber bending, or the fiber tapering [92], as it is depicted in Figure 11. These sensors are usually fabricated onto multimode plastic cladding silica fiber, using a transmission architecture, which simplifies the optical set-up.

In these cases, when using optical fiber as a waveguide, evanescent wave absorbance-based techniques are employed [93,94]. In order to enhance the interaction between the light and the sensing elements, different parameters of the optical instrumentation can be adjusted, such as the fiber diameter, the bending radius, the numerical aperture or the working wavelength [95].

By means of a U-bent PMMA fiber probe in order to increase the penetration depth, a label-free optical fiber biosensor platform was developed and tested against Goat anti-human IgG (GaHIgG) [96] and *E. coli* cells [97,98]. First, after analyzing the relationship between the fiber diameter and the absorbance (the second increased with the decrease in the first), a 5 cm-length section of a 200 μm-core optical fiber was uncladded and U-bent. Then, it was functionalized (with either Human IgG antibodies (HIgG) or monoclonal antibodies against *E. coli*) and illuminated with an LED centered at 280 nm, in order to measure absorbance at that wavelength. When immobilizing HIgG onto the optical fiber for the detection of GaHIgG, the absorbance increased as the analyte concentration did. In particular, the sensor was able to detect GaHIgG from 0.1 to 50 μg/mL, with an LOD of 0.1 μg/mL GaHIgG.

The utilization of metallic NPs allows the development of LSPR-based sensors, which offer the advantage of monitoring both absorbance intensity and wavelength shift [99]. Furthermore, these sensors work in the visible range, which also cheapens the cost of the optical instrumentation. For the detection of GaHIgG, Punjabi et al. [100] labelled both the antibody and the antigen with AuNPs of different sizes: an LSPR centered at 535 nm was generated when immobilizing the AuNPs-tagged HIgG onto the optical fiber, whose intensity increased and wavelength was red-shifted in the presence of AuNPs-labelled GaHIgG. The dynamic range was from 0.5 to 10 μg/mL. In the case of [101], an optical fiber sensor for the detection on interleukin-1β (IL-1β) in synovial fluids is reported: it is based on Au NPs modified by a self-assembled monolayer of 6-mercapto-1-hexanol (MCH) and 11-mercaptoundecanoic acid (MUA), functionalized with anti-IL-1β antibodies. The sensor was capable of detecting IL-1β concentrations in the range from 4.98 × 10^−11^ M to 9.95 × 10^−9^ M, with a LOD of 1.2 × 10^−12^ M and a sensitivity of 5.5 × 10^10^ AU/M.

**Figure 12:**
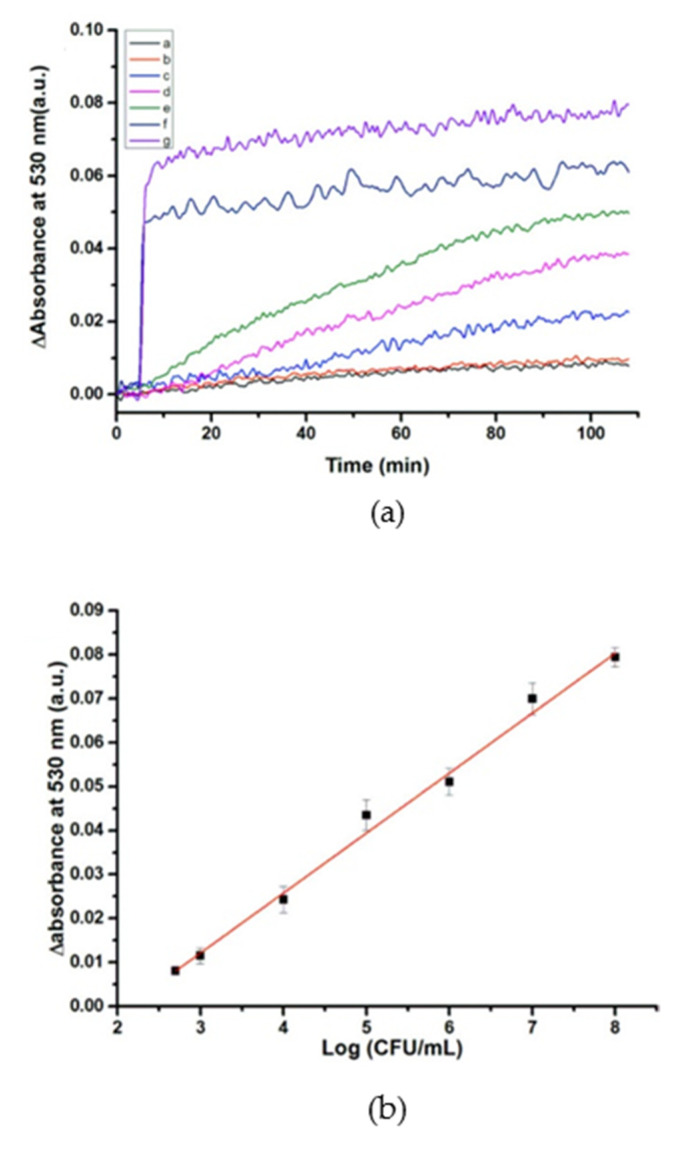
(**a**) Absorbance variations at 530 nm for (a) 5 × 10^2^ CFU/mL, (b) 1 × 10^3^ CFU/mL, (c) 1 × 10^4^ CFU/mL, (d) 1 × 10^5^ CFU/mL, (e) 1 × 10^6^ CFU/mL (f) 1 × 10^7^ CFU/mL, and (g) 1 × 10^8^ CFU/mL concentrations of *S. typhimurium*. (**b**) Linear relationship between the variation of the absorbance at 530 nm and the concentration of *S.* *typhimurium* (logarithmic scale). Reprinted with permission from [102].

Another example reporting the utilization of AuNPs for the development of an LSPR-based optical fiber biosensor is that presented in [102] by Xu et al. After comparing different fiber shapes in order to enhance the sensitivity towards refractive index variations, the sensor was developed onto a 600 μm-core Ω-bent fiber by functionalizing with 3-APTMS and AuNPs. Then, DNA strands for the detection of *S. typhimurium* were immobilized on the AuNPs. As is shown in Figure 12, the absorbance of the sensor increased as the concentration of the target bacteria did. The sensor performance was analyzed in a Tris-HCl buffer (pH 7.4), presenting an LOD of 128 CFU/mL, a dynamic range which varied from 5 × 10^2^ to 1 × 10^8^ CFU/mL and a sensitivity of 0.013 AU/log(CFU/mL), and it was also capable of detecting *S. typhimurium* in chicken samples. Furthermore, it was tested against other bacteria, such as *S. aureus*, *E. coli*, *S. enteritis* and *Shigella*, without any cross-sensitivity.

AuNPs functionalization with glucose oxidase (GOx) can be used to fabricate LSPR-based glucose biosensors, as the enzyme allows the real time detection of the target analyte [103]. The device presented in [104], based on a U-bent fiber, exhibited a decrease in the absorbance at 540 nm with the increase in the glucose concentration, as a consequence of the change in the polarization properties of the AuNPs. Furthermore, the authors analyzed the effect of the bending radius of the sensor onto its sensitivity for a given glucose concentration of 100 mg/mL, concluding that the optimal bending radius was 0.982 mm.

K. Li et al. [105], who developed a reversible optical fiber biosensor for the detection of a cancer biomarker (alpha-fetoprotein) in serum, also studied the relationship between the dimensions of the fiber and the Au NPs: their calculations indicated that, when using an unbent fiber, the sensitivity of the sensor could be enhanced by decreasing the diameter of the fiber or by increasing the size of the Au NPs.

**Figure 13:**
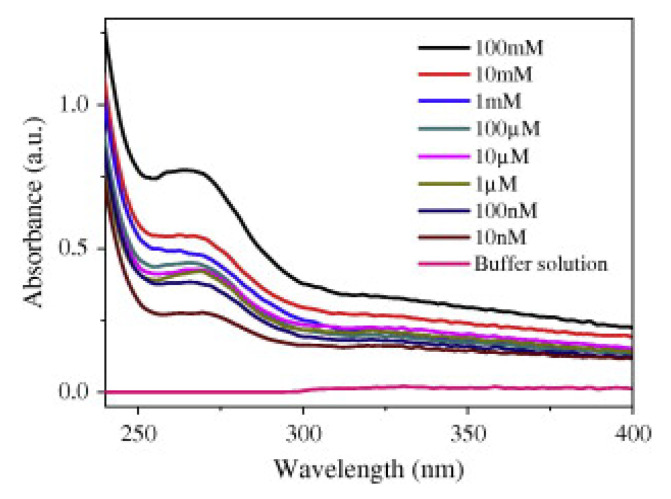
Absorbance spectra of the sensor for glucose concentrations in the range from 10 nM to 100 mM. Reprinted with permission from [107].

Apart from metallic NPs [104], polymeric thin films [106] can also be functionalized with GOx. For instance, Pahurkar et al. [107] immobilized GOx through cross-linking via 1% glutaraldehyde onto a polyaniline (PANI) layer that had been previously in-situ deposited onto the fiber core for the development of an EWA-based glucose biosensor. The interaction between GOx and glucose enhanced the π-π* transitions in PANI, producing absorption peak at 272 nm: as the glucose concentration of the samples increased, so did the absorbance at that wavelength, as it is depicted in Figure 13. The sensor responded to glucose concentrations from 10 nM to 100 nM, presenting an LOD of 10 nM in 0.1 M PBS. Besides, it exhibited a stable time response, performing the same 36 days after its fabrication.

Apart from the applications described in this section, many others, such as vitamin A, taurine or urea detection, have been carried out during recent years. They are summarized in the following tables. Just for the sake of clarity, these tables have been classified according to the bioreceptors employed: IgG or HIgG antibodies (Table 5), other antibodies (Table 6), enzymes (Table 7) and other bioreceptors (Table 8).

## 5. Conclusions

This comprehensive review has analyzed the main luminescence and absorption-based optical fiber biosensors that the scientific community has published in the literature during the last decade. The main objective has been to discover what kind of optical fibers and configuration set-ups are normally utilized, the main parameters to consider when fabricating and characterizing them and what applications they are used for. After all, the following lines summarize the main conclusions that arose and the trends in the state of the art when designing this kind of biosensors.

From an optical point of view, most of the biosensors based on intensity measurements are designed in the UV-VIS range, using thick diameter multimode fibers and with simple configuration set-ups based typically on an LED, a bifurcated fiber and a photoreceptor. The fact of working with this kind of equipment is, surely, a good choice for real applications. An investment to implement these set-ups should not be an issue.

In the case of luminescence-based biosensors, they are built on multimode fibers, which are often tapered in order to achieve a better light coupling from the fluorophores to the fiber waveguide. Here, the reflection set-up is the most used since it facilitates the light coupling from the luminescent molecules to the fiber core. Most of the reported contributions show that luminescence can be detected as long as the Stokes shift between excitation and emission peaks is higher than 20 nm. Moreover, the detections can be direct or indirect, depending on whether the luminophore is the bioreceptor directly or whether it indicates a higher or lower number of bioreceptor-analyte interactions. There is a wide range of potential applications for luminescent biosensors that can cover from the detection of DNA strands to that of water pollutants with very high sensitivity and low LOD.

Regarding absorption-based biosensors, several sensing schemes have been described in order to enhance the light coupling from the sensing film towards the fiber core. Unlike luminescence techniques, a great part of the reported set-ups are in transmission, even using bent fibers to couple as much light as possible to the evanescent wave. The typical absorption wavelengths are located between 500 and 650 nm, due to the use of gold nanoparticles mainly. Although some analytes can be detected by their specific absorption at certain wavelengths, most of them are monitored thanks to the utilization of antibodies deposited onto metallic nanoparticles, directly transducing the biointeractions into light absorption. Taking into account both the wavelength ranges and the possibility of working with metallic nanoparticles, fabricating LSPR-based biosensors seems to be the most common strategy when developing absorption measurements.

To sum up, the evidence shown within this review reveals the great potential of intensity-based optical fiber biosensors. Different types of biocompatible fibers; multiple sensing schemes and optical configurations to be utilized and adapt to different experimental requirements; a wide variety of bioreceptors and the possibility of labelling them with fluorophores or metallic nanoparticles. These facts, and the possibility of dealing with colorimetric techniques already used in clinical analytics and even in point-of-care applications, render this technology of high consideration and an interesting alternative for the development of simple and cost-effective future biomedical devices.

## Figures and Tables

**Table 1:** Optical fiber biosensors based on luminescence using DNA strands or aptamers as bioreceptors for the detection of DNA strands. The dash indicates that information about that parameter is not available in the original manuscript.

Target Analyte	Bioreceptor	OF Configuration	Stokes Shift (λabs–λem)	LOD	Dynamic Range	Sensitivity (S) (Slope)	Detection Media	Regeneration	Cross-Sensitivity	Ref.
Quantum Dots-labelled DNA strand	DNA strand	600 µm-core tapered OF (Rx)	300 nm (405 nm–705 nm)	3.2 × 10^−18^ M	1 × 10^−10^ M–2.5 × 10^−9^ M	-	PBS buffer (10 mM, pH 7.4)	SDS (pH 1.9)	-	[31]
DNA strand	Fluorophore-labelled DNA	480 µm-core tapered OF (Rx)	25 nm (644 nm–669 nm)	5.7 × 10^−10^ M	1 × 10^−8^ M–1 × 10^−5^ M	55 AU/M from 10^−8^ M to 10^−6^ M 550 AU/M from 10^−6^ M to 10^−5^ M	Tris-HCl buffer (10 mM, pH 8)	2mM HCl	-	[29]
DNA strand	Oligonucleotide strands	45mm of 400 µm-core OF (Tx)	Green QDs: > 121 nm (407 nm–528 nm) Red QDs: > 211 nm (407 nm–618 nm)	-	1 × 10^−8^ M–2 × 10^−8^ M	-	Tris-borate buffer pH 7.4	-	No	[35]
DNA strand (Shigella)	Biotin-labelled DNA strand	600 µm-core tapered OF (Rx)	20 nm (683 nm–703 nm)	10^−10^ M	0–2.5 × 10^−9^ M	931.14 AU/nM	20 mM Tris–HCl, pH 8.0, 0.5 M MgCl2	0.5% SDS (pH 1.9)	-	[74]
DNA strands of Aminoglycoside antibiotics (AMGA)	DNA strand	600 µm-core diameter (Rx)	Cy3: 14 nm (554 nm–568 nm) Cy5: 17 nm (649 nm–666 nm)	2.6 × 10^−8^ M	2 × 10^−7^ M–2 × 10^−4^ M	0.0278 AU/µM	Tris-HCl buffer (10 mM), milk products	SDS solution	No	[75]

**Table 2:** Optical fiber biosensors based on luminescence using DNA strands or aptamers as bioreceptors for the detection of analytes different from DNA strands. The dash indicates that information about that parameter is not available in the original manuscript.

Target Analyte	Bioreceptor	OF Configuration	Stokes Shift (λabs–λem)	LOD	Dynamic Range	Sensitivity (S) (Slope)	Detection Media	Regeneration	Cross-Sensitivity	Ref.
*Escherichia coli (E. coli) O157:H7*	DNA strand	500 µm-core fiber (Tx)	20 nm (683 nm–703 nm)	Lower than 10 CFU/mL	10–10^5^ CFU/mL	−12.557 (%/(CFU/mL)) at 37 °C. −10.796 (%/(CFU/mL)) at 30.8 °C	Real waste water samples	5% SDS	Adenovirus, rotavirus and salmonella	[76]
	*E.coli O157: H7* aptamer	225 μm of taper waist (Rx)	20 nm (683 nm–703 nm)	110 CFU/mL	350–3 × 10^6^ CFU/mL	-	Sterilized samples of tap water, effluent of wastewater treatment plant and landscape water	From 80% to 110%	No	[77]
*S. typhimyrium*	*S. typhimyrium* aptamer	225 μm of taper waist (Rx)	20 nm (683 nm–703 nm)	210 CFU/mL	450–7.8 × 10^6^ CFU/mL	−17.96 AU/log(CFU/mL) in blood diluted to a final blood concentration of 10%	Blood diluted to a final blood concentration of 10%	-	No	[77]
Hg^2+^	T-rich oligodeoxyribonucleotide (ON) sequence	600 µm-core diameter (Rx)	20 nm (683 nm–703 nm)	2.1 × 10^−9^ M Hg^2+^ (1 × 10^−8^ M cDNA) 5 × 10^−9^ M Hg^2+^ (2 × 10^−8^ M cDNA)	0–6 × 10^−7^ M (1 × 10^−8^ M cDNA) 0–2 × 10^−6^ M (2 × 10^−8^ M cDNA)	-	MES buffer (0.01 M, pH 7), tap water, bottled water, and a wastewater treatment plant	0.5% SDS solution (pH 1.9)	No	[62]
Hg^2+^	T-rich oligodeoxyribonucleotide (ON) sequence	1 mm-core diameter (Rx)	86 nm (390 nm–476 nm)	4.73 × 10^−13^ M Hg^2+^ in PBS9.03 × 10^−13^ M Hg^2+^ in ultrapure water	5 × 10^−12^ M–5 × 10^−9^ M Hg^2+^ in PBS. 1 × 10^−12^ M–5 × 10^−10^ M Hg^2+^ in ultrapure water. 1 × 10^−12^ M–1 × 10^−10^ M Hg^2+^ in tap water	0.0582 Δ(I_0_/I)/log(Hg^2+^ (M)) in PBS0.0337. Δ(I_0_/I)/log(Hg^2+^ (M)) in ultrapure water. 0.0436 Δ(I_0_/I)/log(Hg^2+^ (M)) in tap water	PBS (0.01, pH 7.4), ultrapure water and tap water	0.5% SDS solution	Negligible interference from heavy metal ions	[51]
Pb^2+^	Cy5.5-labelled DNA strand	600 µm-core diameter (Rx)	20 nm (683 nm–703 nm)	1 nM Pb^2+^	2 × 10^−8^ M–8 × 10^−7^ M Pb^2+^	19.23 × 10^6^ AU/(M Pb^2+^)	0.1 M Na_2_HPO_4_·7H_2_O buffer, 0.2 M NaCl, 0.05% Tween-20, pH 7.2. Also tested in: bottled purified water, tap water, mineral spring water	0.5% SDS (pH 1.9)	No	[37]
Pb^2+^	DNA strand	600 µm-core diameter (Rx)	20 nm (683 nm–703 nm)	1.03 × 10^−9^ M Pb^2+^	2–7.5 × 10^−8^ M (linear 2–5 × 10^−8^ M)	5.721 × 10^9^ AU/M	NaHEPES (0.05 M, pH 7.26), tap water and effluent froms two wastewater treatment plants	1% SDS (pH 1.9), PBS and 1 mg/mL BSA	No	[63]
Pb^2+^	DNA strand	600 µm-core diameter (Rx)	35 nm (490 nm–525 nm)	1.06 × 10^−9^ M Pb^2+^	7.5 × 10^−8^–10^−9^ M	2.45 × 10^9^ NPA/M (NPA—Neat Peak Area)	MOPS buffer (0.01 M, pH 7.5), bottled, tap and pond water	-	No	[78]
Bisphenol A (BPA)	DNA strand	600 µm-core diameter (Rx)	20 nm (683 nm–703 nm)	1.86 × 10^−9^ M (0.45 ng mL) Bisphenol A	2 × 10^−9^ M–100 × 10^−7^ M	-	0.1 M PBS buffer. Also tested in wastewater	0.5% SDS	No	[61]
	Cy3-labelled DNA strand	200 μm-core hollow core anti-resonant fiber (HARF) with 13 μm-hole diameter	14 nm (554 nm–568 nm)	1.69 × 10^−12^ M	1 × 10^−11^ M–1 × 10^−9^ M (linear from 1 × 10^−11^ M to 6 × 10^−10^ M)	1.27 × 10^12^ AU/M (from 1 × 10^−11^ M to 6 × 10^−10^ M)	Blood and environmental samples	1 M urea	No (BPB and BPS studied)	[79]

**Table 3:** Optical fiber biosensors based on luminescence using antibodies as bioreceptors. The dash indicates that information about that parameter is not available in the original manuscript.

Target Analyte	Bioreceptor	OF Configuration	Stokes Shift (λ_abs_–λ_em_)	LOD	Dynamic Range	Sensitivity (S) (Slope)	Detection Media	Regeneration	Cross-Sensitivity	Ref.
*E. coli O157:H7*	Antibodies	780 μm-core polystyrene fiber (Rx)	17 nm (649 nm–666 nm)	1 × 10^3^ cell/mL in buffer and milk	1 × 10^3^–1 × 10^7^ cell/mL in buffer and in milk	-	PBS (pH 7.4, 10 mM) and milk	Ultrapure water	No	[30]
	Antibodies	Borosilicate glass fiber (Tx)	13 nm (627 nm–640 nm)	3.0 × 10^7^ CFU/mL CFU = colony forming unit = number of bacteria	-	-	PBS buffer	-	-	[80]
Estrogen receptor α protein from MCF-7 Breast carcinoma cells and MDA-MB 231 cells	Antibody (antiestrogen α)	Hollow core photonic crystal fiber (core diameter 6 ± 1 µm, cladding diameter of 122 ± 5 µm) (Tx)	Alexa Fluor 488: 35 nm (490 nm–525 nm) Alexa 555: 25 nm (555 nm–580 nm)	20 pg ERα protein in 50 nL sample volume	-	-	Cell lysate	-	-	[57]
Interleukin-6 (IL-6) protein	Antibody	Tapered 600 µm-core OF (Rx)	35 nm (490 nm–525 nm)	5 × 10^−12^ M	5 × 10^−12^ M–1.5 × 10^−10^ M	-	PBS and EA buffer, and serum samples	Ultrapure water	No	[70]
Swine-origin influenza A (H1N1) virus (S-OIV) hemagglutinin (HA) protein	Antibody	1 mm-core OF (Tx)	24 nm (633 nm–657 nm)	S-OIV HA protein: 13.9 pg/mL in PBS S-OIV isolates (original culture): 8.25 × 10^4^ copies/mL in PBS, 1.65 × 10^5^ copies/mL in mimic solution (human nasal mucosa)	5–50 ng/mL S-OIV HA in PBS (linear)	-	PBS and human nasal mucosa	-	No	[71]
Bisphenol A (BPA)	Fluorescence-labeled anti-BPA antibodies	Tapered fiber: 225 µm-core, 15 mm length (Rx)	20 nm (683 nm–703 nm)	2.63 × 10^−10^ M	2.19 × 10^−9^ M–4.38 × 10^−7^ M	4.88 × 10^7^ AU/M	PBS (0.01 M, pH = 7.4)	0.5% SDS (pH 1.9)	BPB	[81]
2,4-Bisphenol-A (BPA) and 2,4-Dichlorophenoxyacetic acid (2,4-D)	Cy5.5 labeled anti-2,4-D antibody and Pacific Blue dye labeled anti-BPA antibody	600 µm-core diameter (Rx)	Cy5.5: 20 nm (683 nm–703 nm) Pacific blue: 45 nm (410 nm–455 nm)	BPA: 2.98 × 10^−11^ M 2,4-D: 1.45 × 10^−11^ M	BPA: 7.45 × 10^−11^ M–5.36 × 10^−7^ M 2,4-D: 4.07 × 10^−11^ M–4.56 × 10^−7^ M	6.99 × 10^8^ M^−1^ (BPA) 6.15 × 10^8^ M^−1^ (2,4—D)	PBS (0.01 M, pH = 7.4) Also tested in real water	0.5% SDS (pH 1.9)	-	[82]
Atrazine and 2,4-D	Fluorophore-labelled antibodies	600 µm-core diameter (Rx)	20 nm (683 nm–703 nm)	Atrazine: 1.4 × 10^−10^ M 2,4-D: 1.81 × 10^−10^ M	Atrazine: 6.95 × 10^−10^ M–5.03 × 10^−7^ M 2,4-D: 4.52 × 10^−10^ M–4.64 × 10^−7^ M	Atrazine: -7.3 × 10^5^ AU/M 2,4-D: -9.95 × 10^5^ AU/M	PBS (0.01 M, pH = 7.4) Also tested in lab tap water and water from natural sources	0.5% SDS (pH 1.9)	-	[83]
Microcystin-LR (MC-LR)	Cy5.5-labelled anti-MC-LR antibody	600 µm-core quartz fiber (Rx)	20 nm (683 nm–703 nm)	4.02 × 10^−11^ M	9.04 × 10^−11^ M–1.1 × 10^−7^ M	-	PBS (0.01 M, pH 7.4) and real water samples	0.5% SDS (pH 1.9)	Negligible	[73]
2,4-Dichlorophenoxyacetic acid (2,4-D)	Cy5.5-labelled anti-2,4-D antibody			4.07 × 10^−10^ M	8.14 × 10^−10^ M–4.51 × 10^−7^ M	-				
Atrazine (ATZ)	Cy5.5-labelled anti-ATZ antibody			9.27 × 10^−11^ M	1.85 × 10^−10^ M–5.12 × 10^−7^ M	-				
Bisphenol A (BPA)	Cy5.5-labelled anti-BPA antibody			1.31 × 10^−10^ M	2.19 × 10^−10^ M–4.82 × 10^−7^ M	-				
Cholylglycine (CG)	Cy5.5-labelled CG antibody	220 µm-core diameter (Rx)	20 nm (683 nm–703 nm)	5.37 × 10^−8^ M	1.07 × 10^−7^ M–1.07 × 10^−5^ M (linear from 1.52 × 10^−7^ M to 2.15 × 10^−6^ M)	2.9 × 10^8^ AU/M	PBS (pH 7.4) and serum samples	0.5% SDS (pH 1.9)	No	[84]

**Table 4:** Optical fiber biosensors based on luminescence using bioreceptors different from DNA strands, aptamers and antibodies. The dash indicates that information about that parameter is not available in the original manuscript.

Target Analyte	Bioreceptor	OF Configuration	Stokes Shift (λ_abs_–λ_em_)	LOD	Dynamic Range	Sensitivity (S) (Slope)	Detection Media	Regeneration	Cross-Sensitivity	Ref.
Glucose	fluorescently labeled glucose-binding lectin concanavalin A (Con A) and dextran	500 µm-core polymer OF (Rx)	Not specified	-	-	-	-	-	-	[85]
	BADAN-labelled Green Binding Protein (GBP)	1.96 mm-core OF (Rx)	120 nm (400 nm–520 nm)	-	4 × 10^−6^ M–2 × 10^−5^ M (linear)	2.3 × 10^3^ AU/M	Ultrapure water	PBS buffer, ultrapure water	-	[58]
	BADAN-labelled GBP	1.96 mm-core OF (Rx)	120 nm (400 nm–520 nm)	2 × 10^−6^ M in PBS Yucatan minipig skin	4 × 10^−6^ M–2 × 10^−5^ M (linear) in PBS	1.3 × 10^6^ AU/μM in PBS	PBS (0.1 M, pH 7.4) and Yucatan minipig skin	PBS buffer	-	
Noradrenaline	[Ru(bipy)_3_]^2+^	Fiber dimensions not specified (Rx)	160 nm (450 nm–610 nm)	4.6 × 10^−7^ M	2.4 × 10^−6^–9.2 × 10^−5^ M (linear)	~33.09 × 10^3^ rad/M	Solution at pH 7.0 and rabbit serum	-	-	[86]
Ca^2+^	Yellow Fluorescent Protein (YFP)	100/100 µm core/cladding diameter (Rx)	14 nm (513 nm–527 nm)	-	2.5 × 10^−10^ M–1 × 10^−5^ M	1.47 × 10^6^ CL/M	0.02 M Tris–HCl (pH 7.9)	-	pH	[59]
Cu^2+^	Chimeric metal-binding green fluorescent protein (His6GFP)	Fiber dimensions not specified (Rx)	113 nm (396 nm–509 nm)	-	5 × 10^−7^ M–5 × 10^−2^ M	-320 U/log[Cu^2^^+^ (M)]	0.05 M Na2HPO4 and 300 mM NaCl buffer	EDTA 50 mM, HEPES buffer	No	[87]
17β-estradiol	17β-estradiol aptamer	600 µm-core diameter (Rx)	20 nm (683 nm–703 nm)	2.1 × 10^−9^ M	5 × 10^−9^–75 × 10^−9^ M	-	Tris–HCl (0.01 M, pH 8.0) and wastewater treatment effluent samples	0.5% SDS (pH 1.9)	No	[69]
Nitric oxide	diaminobenzozcridine (VDABA)	Dimensions not specified (Rx)	Not specified, λ_em_ = 492 nm	-	1.8 × 10^−6^–9 × 10^−6^ M (linear)	3.8889 (M NO)	Gas	-	-	[88]
Ethanol	alcohol dehydrogenase enzyme	1.6 mm-core diameter (Rx)	130 nm (360 nm–490 nm)	-	1–3100 ppb	-	Skin gas	-	1-propanol	[89]
1,2-dibromoethane and 3-chloro-2-(chloromethyl)-1-propene	purified enzyme haloalkane dehalogenase and a fluorescence pH indicator	1 mm-core PMMA fiber (Rx)	22 nm (495 nm–517 nm)	1,2-dibromoethane: 0.133 × 10^−3^ M. 3-chloro-2-(chloromethyl)-1-propene: 1.4 × 10^−5^ M	0–1.2 × 10^−6^ M and 0–8 × 10^−7^ M	110.3990 (V/M) (1,2-dibromoethane). 61.0072 (V/M) (3-chloro-2-(chloromethyl)-1-propene)	HEPES buffer (0.001 M, pH 8.2)	-	-	[90]

**Table 5:** Optical fiber biosensors based on absorption using IgG or HIgG antibodies as bioreceptors. The dash indicates that information about that parameter is not available in the original manuscript.

Target Analyte	Bioreceptor	OF Configuration	λ_abs_	LOD	Dynamic Range	Sensitivity (S) (Slope)	Detection Media	Regeneration	Cross-Sensitivity	Ref.
Goat anti-human Ig G (GaHIgG)	Human immunoglobulin G (HIgG) antibody	200 µm-core (U-bent, Tx)	495 nm (FITC) and 530 nm (AuNPs)	-	FITC-GaHIgG: 1.38 × 10^−5^ M–6.95 × 10^−5^ M. AuNPs–GaHIgG: 1.38 × 10^−5^ M–6.95 × 10^−5^ M	-	PBS	-	-	[108]
	Human IgG antibody	200, 400 and 600-μm (U-bent, Tx)	280 nm	6.7 × 10^−10^ M	6.7 × 10^−10^ M–3.35 × 10^−8^ M	-	5 mg/mL BSA solution	-	-	[96,97,98]
	HIgG immobilized onto AuNPs	200 µm-core fiber (U-bent, Tx)	535-548 nm	-	3.47 × 10^−6^ M–6.95 × 10^−5^ M	-	PBS (pH 7.4)	-	-	[100]
	HIgG antibodies	400 μm core PCS fiber (S-shape, Tx)	500 nm	1.7 × 10^−9^ M	1.7 × 10^−9^ M–6.8 × 10^−8^ M	-	PBS (pH 7.4)	-	-	[109]
Human Ig G (HIgG)	GaHIgG antibodies	200 µm-core fiber (U-bent, Tx)	530 nm	2 × 10^−12^ M	6.67 × 10^−12^ M–6.67 × 10^−7^ M	0.019 *A*_530nm_/(log(M) − 11)	PBS (pH 7.4)	-	-	[110]
	Bioreceptors for HIgG (Fab-GaHIgG)	200 μm-core (U-bent, Tx)	530 nm	6.67 × 10^−15^ M IgG using immunogold labels. 6.67 × 10^−9^ M HIgG using immunogold labels and subsequently silver enhancement	6.67 × 10^−15^ M–6.67 × 10^−12^ M IgG	0.04 Abs/(log(M) − 11) using immunogold labels. 0.8 Abs/(log(M) − 11) using immunogold labels and subsequently silver enhancement	PBS	-	-	[94]
	AuNPs functionalized with GaHIgG antibodies	200 μm-core (U-bent, Tx)	530 nm	7 × 10^−18^ M	7 × 10^−18^ M–7 × 10^−12^ M	0.1036 Abs @530 nm/log (M)	PBS buffer	-	-	[111]
	LEEH caped AuNPs	250 μm-core PMMA fiber (U-shape, Tx)	650 nm	12.7 × 10^−6^ M	-	-	MilliQ water	-	-	[112]
Cu^2+^	Human immunoglobulin G (HIgG)	200 μm-core fiber (U-bent, Tx)	650 nm	7.5 × 10^−15^ M in tap water	10^−14^–10^−6^ M	Tap water: 0.006 (Abs @530 nm)/ln[Cu^2+^ (M)]	Tap water, natural water bodies and soil	90–115% (not specified how)	Negligible response to 10 μM of other ions	[113]
Mannosylated Lipoarabinomannan (Mtb LAM)	AuNPs functionalized with anti-Mtb LAM immunoglobin M (IgM) and Anti-Mtb LAM IgG	200 μm-fused silica fiber (U-bent, Tx)	540 nm	5.9 × 10^−9^ M (in PBS buffer) 5.9 × 10^−8^ M (in synthetic urine)	2.95 × 10^−8^ M–5.9 × 10^−5^ M in PBS buffer 5.9 × 10^−8^ M–5.9 × 10^−5^ M in synthetic urine	0.078 (Abs @542 nm/(log[LAM(M)] − 8.3) in PBS buffer 0.043 (Abs @542 nm/log (log[LAM(M)] − 8.3) in synthetic urine	PBS buffer and synthetic urine	-	-	[114]

**Table 6:** Optical fiber biosensors based on absorption using other antibodies than IgG or HIgG as bioreceptors. The dash indicates that information about that parameter is not available in the original manuscript.

Target Analyte	Bioreceptor	OF Configuration	λ_abs_	LOD	Dynamic Range	Sensitivity (S) (Slope)	Detection Media	Regeneration	Cross-Sensitivity	Ref.
*E. coli* O55	*E. coli* antibodies	1 mm-core PMMA fiber (U-bent, Tx)	600 nm and 845 nm	1.5 × 10^3^ CFU/mL	1 × 10^3^–1 × 10^8^ CFU/mL	-	Ultrapure water with NaCl	-	No	[115]
Bovine Serum Albumin (BSA)	BSA antibody	8 μm core/125 μm cladding silica fiber (Tx)	1558 nm	-	-	-	PBS	-	-	[116]
	LEEH caped AuNPs	250 μm-core PMMA fiber (U-shape, Tx)	650 nm	3 × 10^19^ M	-	-	MilliQ water	-	-	[112]
Interleukin-1β	Anti-IL-1β	250 μm-core PCS fiber (Tx)	532 nm	1.2 × 10^−12^ M	4.98 × 10^−11^ M–9.95 × 10^−9^ M	5.5 × 10^10^ AU/M	PBS (pH 7.4)	-	No	[101]
Alpha feto-protein	Alpha feto-protein antibody	600 μm-core PCS fiber (U-bent, Tx)	550 nm	7.33 × 10^12^ M in PBS and 2.85 × 10^13^ M in human serum	4.31 × 10^13^ M–1.72 × 10^15^ M in PBS and human serum	1.24 AU/RIU	PBS and human serum	-	Slight interference from HSA and human IgG	[117]
	Alpha feto-protein antibody	8 μm-core SMF fiber (Tx)	532 nm	1.72 × 10^12^ M in PBS and 1.72 × 10^13^ M in BSA	1.72 × 10^12^ M–8.6 × 10^15^ M)	-	PBS and BSA	0.1 M glycine–HCl buffer (pH 2.3)	-	[105]
Procalcitonin	Procalcitonin (PCT) antibodies	OF dimensions not specified (Tx)	520 nm	3.96 × 10^12^ M	4.17 × 10^13^ M–4.17 × 10^15^ M	0.002 (ΔI/I_0_)/log[PCT(M)]	PBS (pH 7.3)	-	-	[118]

**Table 7:** Optical fiber biosensors based on absorption using enzymes as bioreceptors. The dash indicates that information about that parameter is not available in the original manuscript.

Target Analyte	Bioreceptor	OF Configuration	λ_abs_	LOD	Dynamic Range	Sensitivity (S) (Slope)	Detection Media	Regeneration	Cross-Sensitivity	Observations	Ref.
Blood glucose	Glucose oxidase (GOx)	600 μm-core fiber (U-shape, Tx)	540 nm	1.38 × 10^−5^ M	0–1.38 × 10^−2^ M	S (= A_water_ − A_sample_ at 540 nm for 5 × 10^−3^ M of glucose) depends on the beding radius: S (r = 0.4 mm) = 0.0008 S (r = 0.5 mm) = 0.0016 S (r = 0.65 mm) = 0.0025 S (r = 0.7 mm) = 0.003 S (r = 1 mm) = 0.005 S (r = 1.7 mm) = 0.004	Millipore^®^ water	Distilled water (reused up to 4 times in a month)	-		[104]
Glucose	Glucose oxidase (GOx)	450 μm-core PCS fiber (Tx)	272 nm	1 × 10^−9^ M	1 × 10^−8^ M–1 × 10^−4^ M	-	PBS (0.1 M, pH 7.4)	-	No		[107]
	-	OF dimensions not specified (Tx)	581 nm	-	3.31 × 10^−3^ M–1.38 × 10^−2^ M	7.6 AU/M	Blood serum	-	-	Proof-of-concept	[119]
Taurine	Taurine dioxygenase enzyme	600 µm-core fiber (Tx)	585 nm	5.3 × 10^−5^ M	0–1 × 10^−3^ M	19 AU/M	PBS (0.1 M, pH 7.4)	-	No		[120]
Urea	Enzyme-urease	1000 μm-core PCS fiber (Tx)	250 nm	1 × 10^−7^ M	1 × 10^−7^ M–1 M	-	PBS (0.1 M, pH 7.4)	-	No		[121]
	Enzyme-urease	1000 μm-core PCS fiber (Tx)	245 nm	-	-	-	PBS (0.1 M, pH 7.4)	-	-	Proof-of-concept. Only 1 μM, 10 μM and 100 μM tested	[122]
	Enzyme-urease	400 μm-core PCS fiber (Tx)	250 nm	1 × 10^−8^ M	1 × 10^−8^ M–1 M	-	PBS (0.1 M, pH 7.4)	-	No		[123]
Uric acid	Uricase enzyme	9 μm-core fiber	513 nm	6.56 × 10^−5^ M	1 × 10^−5^ M–1 × 10^−3^ M	−2.1 × 10^3^%/M	PBS (0.1 M, pH 7.4)	PBS (0.1 M, pH 7.4)	No		[124]

**Table 8:** Optical fiber biosensors based on absorption using bioreceptors different from antibodies and enzymes. The dash indicates that information about that parameter is not available in the original manuscript.

Target Analyte	Bioreceptor	OF Configuration	λ_abs_	LOD	Dynamic Range	Sensitivity (S) (Slope)	Detection Media	Regeneration	Cross-Sensitivity	Observations	Ref.
*E. coli* B40 (bacteriophage T4)	B40 cells	200 μm-core (U-bent, Tx)	610 nm		-	5.05 ∆A660nm/RIU (Sensitivity to *E. coli* B40 not studied)	PBS	-	No		[125]
DNA strand	ON sequence	600 µm-core fiber (PMMA) (U-bent, Tx)	535 nm	2 × 10^−10^ M	-	-	PBS	-	-		[93]
*S. typhimurium*	DNA strand	600 μm-core PCS fiber (Ω-bent, Tx)	530 nm	128 CFU/mL	5 × 10^2^ to 1 × 10^8^ CFU/mL	0.013 AU/log(CFU/mL)	PBS (0.1 M, pH 7.4)	93–123% with PBS (0.1 M, pH 7.4)	No		[102]
Vitamin A	Au@Ag core-shell nanoparticles embedded SiO_2_-TiO_2_-ZrO_2_ ternary matrix	600 μm-core PCS fiber (Tx)	400 nm and 500 nm	1 × 10^−5^ M	1 × 10^−6^ M–1 × 10^−2^ M	-	Aqueous solution	-	No		[126]
Concanavalin A	Glycoprotein ribonuclease B (RNase B)	400 μm-core PCS fiber (Tx)	595 nm	-	5.78 × 10^17^ M–4.64 × 10^18^ M	394.56%/RIU (Sensitivity to Con A not studied)	PBS (0.01 M, pH 7.4)	8.0 M urea solution	-	Proof-of-concept	[127]

## Data Availability

Data is contained within the article.
